# Recombinant infectious bronchitis virus containing mutations in non-structural proteins 10, 14, 15, and 16 and within the macrodomain provides complete protection against homologous challenge

**DOI:** 10.1128/jvi.01663-24

**Published:** 2025-02-27

**Authors:** Sarah Keep, Katalin Foldes, Guilia Dowgier, Graham Freimanis, Chandana Tennakoon, Shafiyeel Chowdhury, Adam Rayment, James Kirk, Trinity Bakshi, Phoebe Stevenson-Leggett, Yana Chen, Paul Britton, Erica Bickerton

**Affiliations:** 1 The Pirbright Institute 111636 , Pirbright, United Kingdom; University of Kentucky College of Medicine, Lexington, Kentucky, USA

**Keywords:** infectious bronchitis virus, vaccine, macrodomain, coronavirus, avian, Mac1, attenuation

## Abstract

Infectious bronchitis virus (IBV) is the etiological agent of infectious bronchitis, an acute highly contagious economically important disease of chickens. Vaccination uses live attenuated vaccines (LAVs) that are generated via serial passage of a virulent field isolate through embryonated hens’ eggs, typically 80–100 times. The molecular basis of attenuation is unknown and varies with each attenuation procedure. To investigate specifically targeted attenuation, we utilized reverse genetics to target the macrodomain 1 (Mac1) domain within non-structural protein 3 of the virulent M41 strain. Macrodomains are found in a variety of viruses, including coronaviruses, and have been associated with the modulation of the host’s innate response. Two recombinant IBVs (rIBVs) were generated with specific single point mutations, either Asn42Ala (N42A) or Gly49Ser (G49S), within the Mac1 domain generating rIBVs M41K-N42A and M41K-G49S, respectively. Replication *in vitro* was unaffected, and the mutations were stably maintained during passaging *in vitro* and *in ovo*. While M41K-N42A exhibited an attenuated phenotype *in vivo*, M41K-G49S was only partially attenuated. The attenuated *in vivo* phenotypes observed do not appear to be linked to a reduction in viral replication and additionally M41K-N42A highlighted the N42A mutation as a method of rational attenuation. Vaccination of chickens with either rIBV M41K-N42A or a rIBV containing the Mac1 N42A mutation and our previously identified attenuating Nsp10 and 14 mutations, Pro85Leu and Val393Leu respectively, offered complete protection from homologous challenge. The presence of multiple attenuating mutations did not appear to negatively impact vaccine efficacy.

**IMPORTANCE:**

Infection of chickens with the *Gammacoronavirus* infectious bronchitis virus (IBV) causes an acute respiratory disease, resulting in reduced weight gain and reductions in egg laying making it a global concern for poultry industries and food security. Vaccination against IBV uses live attenuated viruses (LAVs), generated by multiple passages of a virulent virus through embryonated hens’ eggs. The molecular basis of attenuation is unknown and unpredictable requiring a fine balance between loss of virulence and vaccine efficacy. In this study, we targeted the macrodomain of IBV for rational attenuation demonstrating a single point mutation can result in loss of pathogenicity. An IBV vaccine candidate was subsequently generated containing three specific attenuating mutations, to reduce the risk of reversion, which completely protected chickens. The targets in this study are conserved among IBV strains and the coronavirus family offering a potential method of rational attenuation that can be universally applied for vaccine development.

## INTRODUCTION

Infectious bronchitis virus (IBV), commonly referred to as avian coronavirus, is a *Gammacoronavirus* that infects domestic fowl and is the etiological agent of infectious bronchitis (IB). IB is an acute respiratory disease that is typified by snicking, watery eyes, nasal discharge, tracheal rales, and loss of tracheal ciliary activity (1–3). The IBV genome consists of a ~27 kb single strand of positive sense RNA that is capped at the 5′ end and polyadenylated at the 3′ end (4, 5). The first two-thirds of the genome encode the non-structural proteins (nsp), 2–16, and the last third encodes the structural proteins in the order Spike (S)–Envelope (E)–Membrane (M), and Nucleocapsid (N). Interspersed among the structural genes are the accessory genes, 3, 4b, 5, and 7 (3, 6–9). There are several strains of IBV which are classified by both serotype and genotype, with the latter based on the S1 sequence of the S gene (10). Different strains can inflict varying severity of disease with strains such as M41 inflicting classical respiratory disease and nephropathogenic strains such as B1648 and QX inflicting more severe disease due to kidney infection (11, 12). While IB clearly presents a welfare issue, IB continues to remain of significant concern for poultry industries worldwide as IBV-infected chickens exhibit reduced weight gain and reduced egg laying in terms of both quantity and quality (6, 13). In addition, IBV renders chickens susceptible to secondary bacterial infections leading to a further impact for poultry farmers. Poultry, both meat and eggs are a major source of protein across the world and therefore IBV continues to present a significant threat to global food security.

Vaccination against IBV is commonly practiced using a mixture of both live attenuated vaccines (LAVs) and inactivated vaccines. The exact vaccination regime is dependent on both the type and age of the bird and the geographical location, as differing strains of IBV are present in different areas of the world (14, 15). Typically, young chicks are vaccinated with LAVs administered via spray or drinking water and often receive multiple vaccinations at regular intervals from hatch, due to poor levels of cross-protection between IBV strains. Layers are then boosted with inactivated vaccines later in life. While the LAVs available do allow for a degree of control, the current process of LAV generation has several disadvantages. Generation of an LAV involves the serial passage of a virulent field isolate through embryonated hens’ eggs to achieve a vaccine that is attenuated yet remains sufficiently immunogenetic in that it can induce a protective immune response; a fine balance needs to be achieved as over-passaging can impact vaccine efficacy (16). The molecular basis of attenuation is unknown and cannot be predicted by relying on random mutations (17). It has been shown that few consensus-level changes are acquired presenting a short route back to virulence (17). In addition, the process is cumbersome and time-consuming as often between 80 and 100 passages are required (16–18). This paired with the unpredictability of attenuation means the process is ill-adapted for reacting to emerging strains. An alternative method of attenuation is therefore required, one that is rational that can be universally applied across the many IBV strains and potentially therefore applied in the event of an emerging strain.

There have been many avenues explored for the next generation of IBV vaccines and vaccines against other coronaviruses in general (19–23). Vaccine-induced immunity is associated with the Spike (S) glycoprotein which has formed the basis of vaccines and therapeutics against zoonotic coronavirus infections in humans such as severe acute respiratory syndrome coronavirus (SARS-CoV), Middle East respiratory syndrome coronavirus (MERS-CoV), and SARS-CoV-2 (24–26). S-based vaccines, either vectored or protein based, have not achieved sufficient vaccine efficacy against IBV infection (27–33). Research has therefore concentrated on using reverse genetics to develop and investigate methods of rational attenuation including the modification of S (34–38), the deletion of accessory genes (39, 40), and targeting of the non-structural proteins (nsps) (41). Our previous research identified two mutations located in Nsp10 and in Nsp14, Pro85Leu and Val393Leu, respectively, that resulted in both an *in vitro* temperature-sensitive replication and an *in vivo* attenuated phenotype (41, 42). Vaccination with a recombinant IBV (rIBV), denoted M41-R, containing these mutations could induce protection against homologous challenge meeting European industry standards (42). A concern for LAVs however is not only reversion, which the clonal origin of rIBVs generated via reverse genetics minimizes as it eliminates “back selection” of a virulent sequence from a population but also recombination. Recombination between vaccine strains has resulted in the generation of a virulent progeny virus (43, 44). This therefore raises the question of whether multiple attenuating mutations that are spatially distributed across the IBV genome are required to reduce the possibility of recombination-induced reversion to virulence.

Located within Nsp3, a large multi-domain protein, is a macrodomain, ~150 amino acids, which has previously been denoted as the X domain or the ADRP domain, as it was originally shown to remove phosphate from ADP-ribose-1″-phosphate therefore having ADP-ribose-1″-phosphatase (ADRP) activity (45–48). Macrodomains are found in a variety of viruses including hepatitis E virus, Venezuelan equine encephalomyelitis virus, and Chikungunya virus (46, 49, 50). They adopt a characteristic globular structure with a mixed α/β/α fold (51). While earlier focus centered on ADRP activity, it is now more widely accepted that viral macrodomains bind poly-ADP ribose (PAR) and mono-ADP ribose (MAR) and act as de-PAR/MARylating enzymes counteracting ADP-ribosylation, a reversible post-translational modification in which one or more ADP ribose molecules are covalently attached to a protein via specific amino acids (52–55). Studies have suggested that de-MARylating activity counteracting host IFN responses may be the more biologically relevant function of the macrodomain during viral infections (50, 52, 54, 56–59).

Both X-ray crystallography and mutagenesis studies on the Mac1 domain from several different coronaviruses including human coronaviruses 229E (HCoV-229E), SARS-CoV, SARS-CoV-2, and Murine Hepatitis Virus (MHV) have identified several residues of interest including a conserved asparagine (Asn) residue in the catalytic pocket (48, 55, 60–63). Mutation of the conserved Asn residue to Ala results in reduced virulence *in vivo* for MHV and SARS-CoV (58, 60, 64) highlighting a potential candidate residue for rational attenuation. The Asn→Ala mutation was shown to reduce catalytic activity (58) (57, 65). Although much of the earlier research focuses on ADRP activity, the same residues identified are translatable to the now widely accepted de-PAR/MARylating activity of the Mac1 (55). It must be noted that much of the research focused on the Mac1 domain of the alpha- and betacoronaviruses with mammalian hosts, and limited research has been completed for those that infect avian species. Crystal structures of the IBV macrodomain however have been determined and highlighted a disparity between the attenuated IBV strain Beaudette and the pathogenic strain M41 (62, 63). Sequence analysis had previously shown IBV M41 contained a triple glycine motif (Gly-Gly-Gly), in line with several other coronaviruses. In the Beaudette strain, the central glycine is replaced with a Serine resulting in the motif Gly-Ser-Gly. A comparison of the Mac1 crystal structures demonstrated that the Gly to Ser change affected the structure of the ADRP-binding cleft (46, 55, 62, 63). Our previous research showed that neither the restoration of the triple glycine motif in Beaudette nor the replacement of the sequence with the equivalent from M41, conferred a pathogenic *in vivo* phenotype. The role of the triple glycine motif in IBV pathogenicity, however, could not be conclusively determined due to the presence of other attenuating mutations/factors, such as the S gene, in the Beaudette genome (38, 66, 67).

This study aimed to further investigate whether the IBV macrodomain is a pathogenicity factor and whether it is a suitable candidate for rational attenuation and generation of a potential IBV vaccine. Using a vaccinia virus reverse genetics system based on the pathogenic M41 strain (41), we modified the IBV Mac1 domain by changing (i) the conserved Asn to an Alanine (Ala) generating the rIBV M41K-N42A; and (ii) the triple glycine motif to resemble the modified triplet in IBV Beaudette, Gly-Ser-Gly, generating the rIBV M41K-G49S. We assessed the replication of the resulting rIBVs *in vitro* and *in vivo* and identified that the rIBV M41K-N42A exhibited an attenuated phenotype *in vivo*. Chickens were vaccinated with either M41K-N42A or with M41R-N42A which contained additional attenuating mutations in Nsp10 and Nsp14, and subsequently challenged with M41-CK. Both rIBVs induced a protective response against challenge, demonstrating that (i) the macrodomain offers an avenue for rational attenuation and (ii) the combination of mutations in the macrodomain alongside attenuating mutations in Nsp10 and Nsp14 did not impact the efficacy of M41R-N42A as a potential vaccine candidate. The presence of three spatially distanced mutations each capable, individually, of attenuation, without jeopardizing vaccine efficacy represents an important step forward in the generation of rationally attenuated vaccine viruses against IBV.

## MATERIALS AND METHODS

### Cells, embryonated eggs, and tracheal organ cultures

Primary chicken kidney (CK) cells were generated from 2- to 3-week-old specific-pathogen-free (SPF) Rhode Island Red (RIR) chickens as previously described (68) by the Cell Culture Unit at The Pirbright Institute (TPI). Chickens raised for the preparation of primary cells and organ cultures were hatched and reared, housed in raised floor pens with enrichment provided, at TPI. Cells were seeded at a density of 0.8 × 10^6^ cells/mL 3 days prior to use; 2 mL per well was seeded for a 12-well plate and 3 mL per well for a six-well plate. All cells were maintained at 37°C, 5% CO_2_, unless otherwise stated. Tracheal organ cultures (TOCs) were prepared from 2- to 3-week-old SPF RIR chickens as previously described (69) and were incubated at 37°C, 7–8 revolutions per hour (h), unless otherwise stated. TOCs were seeded individually in glass tubes 3 days prior to use. Before use, the ciliary activity of each TOC was confirmed to be 100% using a light microscope. Chicken embryonated hens’ eggs were supplied either by the Poultry Production Unit at TPI, Compton site, the National Avian Facility (NARF), The Roslin Institute, or Valo BioMedia Germany; all were incubated at 37°C.

### Viruses

The rIBV M41-K is a pathogenic molecular clone of M41-CK (17) which has been described previously (41). M41-CK is a pathogenic isolate of IBV belonging to the G1-1 genotype and Massachusetts serotype (10). The rIBV M41-R contains four-point mutations, has attenuated *in vivo* phenotype, and a temperature-sensitive replication phenotype; this virus has also been described previously (41, 42). The rIBVs M41K-N42A, M41K-G49S, and M41R-N42A were generated for this study; the first two are based on the M41-K genome and the last on the M41-R genome. All viruses were propagated in 10-day-old SPF embryonated hens’ eggs, with allantoic fluid harvested 24–48 hours post-infection (hpi) and clarified by low-speed centrifugation. All virus stocks were titrated in triplicate by plaque assay in CK cells as previously described (69). All nucleotide and amino acid residues correspond to the M41-CK sequence, accession number MK728875 (17).

### Sequences for investigation of Mac1 conservation

IBV sequences downloaded from GenBank were utilized for the investigation of the conservation of the Mac1 domain. The following full-genome sequences of IBV that were used: Beaudette (AJ311317), H120 (ON350836), M41 (MK937830), Gray (GU393334.1), Armidale (KU556805), Vic-S (KF460437.1), ckAusV590 (MK990812), ck/ch/LHLJ/110664 (MN509589), Ark99 (MH779860.1), 4/91 (KF377577.1), CR88 (MN548285), B1648 (KR231009), D388 (MN54828971), QX (MN548289), Italy02 (MN548288), IBV/Ck/EG/CU/4/2014 (KY805846), GA/1476/2015 (MN599049), D1466 (MN548286), and GX-NN09032 (JX8979001). The *Gammacoronavirus* Turkey Coronavirus (NC_010800.1). Alpha- and betacoronaviruses: MERS-CoV (MK967708.1), SARS-CoV (AY278488.2), SARS-CoV-2 Wuhan-Hu-1 (NC045512.2), SARS-CoV-2 Alpha (OV054768.1), SARS-CoV-2 Beta (OX008586.1), SARS-CoV-2 Delta (OK091006.1), SARS-CoV-2 Gamma (OR578388.1), SARS-CoV-2 Omicron (OL672836.1), HCoV-229E (PP810610), HCoV-NL63 (JX504050.1), HCoV-HKU1 (KF686346.1), HCoV-OC43 (KX344031.1), Bovine coronavirus (LC830477), Canine coronavirus (PP526172.1), Feline Coronavirus (KX722531), MHV (AY700211) and PEDV (MK841495.1). Avian *Deltacoronavirus* sequences used were as follows: Wigeon coronavirus HKU20 (NC016995), White-eye coronavirus HKU16 (NC_016991.1), Thrush coronavirus HKU12 (NC011549.1), Night heron coronavirus HKU19 (NC016994.1), Munia coronavirus HKU13 (NC_011550.1), Common moorhen coronavirus HKU21 (NC016996.1), and Bulbul coronavirus HKU11 (NC_011547.1).

### Generation of recombinant IBV

A vaccinia virus-based reverse genetics system previously described (70, 71) was used to generate the two rIBVs based on the pathogenic clone of M41-CK, M41-K and a final rIBV based on the attenuated rIBV M41-R (41). The first M41K-N42A contains the nucleotide mutations AA_3646-3647_GC resulting in the amino acid mutation N42A within the Mac1 domain. M41K-G49S contained the nucleotide mutation G_3667_A, introducing the amino acid change G49S. The final rIBV based on M41-R contains the nucleotide mutations AA_3646-3647_GC resulting in the amino acid mutation N42A, generating M41R-N42A. All rIBVs were recovered in CK cells as previously described and viral stocks were generated after three (M41K-G49S) or four (M41K-N42A and M41R-N42A) passages in CK cells. The mutations were confirmed by Sanger sequencing.

### Next-generation sequencing

RNA was prepared from 2 to 4 mL of stock virus of M41K-N42A, M41K-G49S, and M41R-N42A by a previously published protocol (72). Quality and quantity of total RNA were measured using the Tapestation RNA screentape (Agilent) and Qubit RNA kit (Life Technologies). A total of 100 nanograms of total RNA from each sample was processed using the RNA prep with an Enrichment kit (Illumina) and a custom panel of hybridization probes (Arbor Daicel) designed to enrich for M41 genomes. Samples were plexed at the hybridization stage with 200 ng per sample. After amplification and elution, hybridized libraries were quantified using a Qubit DNA BR kit (Life Technologies) and Tapestation D1000 (Agilent) using established protocols. Hybridized library was diluted to 2 nM and quantified with qPCR (NEB) prior to being loaded onto a MiSeq micro 300 cycle flow cell and reagent kit with a 1% PhiX spike-in (Illumina).

Fastq was subjected to QC assessment using Fastqc and trimmed using Fastp (73). Reads were aligned using Bwa-mem (74) to the M41-CK reference sequence. Samtools (75) was used to process the files and covert them to Bam files prior to indexing. Bcftools (76) and Ivar (77) were used to generate consensus sequences which were generated to examine differences between viruses and the reference genome.

### Assessment of growth kinetics *in vitro*

CK cells, seeded in six-well plates were infected with 10^4^ PFU per well (~MOI of 0.01) of M41K-N42A, M41K-G49S, or M41-K or 10^4^ PFU of M41R-N42A or M41-R and incubated for 1 h at 37°C or 41°C, 5% CO_2_. Cells were washed twice with PBS to remove any unbound virus and 3 mL of BES (N,N-Bis(2-hydroxyethyl)−2aminoethanesulphonic acid) medium was added per well (71) and incubated at either 37°C or 41°C, 5% CO_2_. Supernatants from one well per plate were harvested at 1 hpi and then every 24 h; the quantity of infectious progeny was determined via titration in triplicate in CK cells (69).

### Assessment of plaque phenotype

CK cells seeded in six-well plates were inoculated in duplicate with 500 µL of the rIBV M41K-N42A, M41K-G49S, and M41-K or in a separate experiment M41-R and M41R-N42A at MOI of 0.1, 0.01, and 0.001. After 1 h incubation at 37°C, cells were washed twice with PBS and BES medium containing 1% agar added. At 96 hpi, cells were fixed with 3.3% formaldehyde and stained with 0.1% crystal violet. Observation of the plaque phenotypes was conducted by visual inspection and measurements of plaque sizes were taken using ImageJ (78), counting 20 or 30 individual plaques from two independent wells.

### Assessment of ciliary activity in *ex vivo* TOCs

In replicates of 10, TOCs were inoculated with 500 µL containing 10^4^ PFU of either M41-K, M41K-N42A, M41K-G49S or in a separate experiment M41-R, M41R-N42A or mock infected with TOC media only (0.5 x EMEM, 75 µM a-methyl-D-glycoside, 40 µM HEPES, 0.1% sodium bicarbonate, 10 U/mL penicillin, and 10 mg/mL streptomycin), and incubated upright at either 37 or 41°C. After incubation, inoculum was removed and replaced with 1 mL media followed by incubation at either 37 or 41°C, rolling at 7 to 8 revolutions per h. The activity of the cilia of each TOC was assessed daily using a light microscope and scored at either 0%, 25%, 50%, 75%, or 100% activity.

### Assessment of growth kinetics in *ex vivo* TOCs

Three TOCs were seeded per glass tube, washed once with PBSa, and inoculated with 500 µL TOC media containing 10^4^ PFU of M41-K, M41K-N42A, or M41K-G49S and in a separate experiment M41-R or M41R-N42A. Infected TOCs were incubated upright for 1 h at either 37 or 41°C after which the inoculum was removed and the TOCs washed twice with PBSa. Per tube, 1 mL TOC media was added and TOCs were incubated at either 37 or 41°C, rolling at 7–8 rev/h. The supernatant was harvested per tube at 1, 24, 48, 72, and 96 hpi and the quantity of infectious progeny was determined by titration, in either duplicate or triplicate in CK cells. Per biological repeat, two technical repeats for each time point were included.

### Assessment of replication *in ovo*

In either duplicate or triplicate, 10-day-old SPF embryonated hen’s eggs from Valo were inoculated with 2 × 10^3^ PFU of M41-K, M41K-N42A, or M41K-G49S or in a separate experiment M41-R or M41R-N42A. Infected eggs were incubated for 24 h at 37°C after which the embryos were culled via refrigeration at 4°C for at least 4 h. The allantoic fluid was harvested and the quantity of infectious progeny was determined via plaque assay in CK cells.

### Serial passaging

#### In ovo

10-day-old SPF RIR were inoculated in triplicate via the allantoic cavity with 0.1 mL of PBS containing a 1:100 dilution of M41K-N42A, M41K-G49S, or M41-K. For passaging of M41R-N42A, 10-day-old SPF eggs from Valo were used and a dilution of 1:200. Eggs were incubated for 24 h at 37°C after which the embryos were culled by refrigeration for at least 4 h. Allantoic fluid was collected, clarified by low-speed centrifugation and used for the next passage following the same protocol.

#### CK cells

Confluent CK cells seeded in six-well plates were washed once with PBS and inoculated in triplicate with 500 µL BES medium containing either M41K-N42A, M41K-G49S or M41-K, at a 1:100 dilution. For passaging of M41R-N42A, a 1:10 dilution was used for the first passage, followed by 1:5 for passages 2–8 and 1:2.5 for passages 9 and 10. Mock-infected wells were inoculated with BES medium. After 1 h incubation at 37°C, the inoculum was removed, and the cells were washed once with PBS followed by the addition of 3 mL BES medium per well. The supernatant was harvested 24–48 hpi depending on observation of cytopathic effect (CPE) and subsequently used for the following passage.

At passage 10 RNA was extracted from 170 µL supernatant or allaontic fluid using RNeasy mini kit (Qiagen), RNA clean-up protocol and analyzed by RT-PCR for the presence of IBV-derived RNA using primers targeting the 3′ untranslated region (UTR) (79) and the Mac1 domain. The sequence of the Mac1 domain, as well as when appropriate the sequence of nsp10 and14, was determined using Sanger sequencing from PCR products generated using the following primer sets: Mac1: For 5′-CTAATTCAGAATGTGAAG-3′ and Rev 5′-GTTCAGCAATAGTTCTCCTT-3′ (1441 nts product), nsp10 For 5′-GGAATGGGCATAATAAGG-3′ and Rev 5′-CGGGTACGGGGTAGCAGTG-3′ (863 nts product) and nsp14 For 5′-CAGACAGTAGACTCGTCTCAAGG-3′ and Rev 5′-GTGCATATACAGACATAGAACC-3′ (2093 nts product).

### *In vivo* experiments

Chickens were housed in raised floor pens in positive-pressure, HEPA-filtered isolation rooms, with each experimental group housed in a separate room. Chickens were provided with enrichment which included soft bedding and/or live feed and/or perches to promote normal animal behavior. All chickens were monitored at least twice daily throughout the duration of the experiment. Humane endpoints for the *in vivo* studies were as follows: (i) sitting alone and not evading capture; the bird will be euthanized immediately, (ii) respiratory distress, for example, excessive gasping; the bird will be euthanized immediately, (iii) rales for 7 days in total; the bird will be euthanized on the beginning of the 7th consecutive day, and (iv) excess drinking for more than two days as indicated by a fluid-filled crop: the bird will be euthanized at the beginning of the 3rd consecutive day.

#### *Experiment 1: Assessment of pathogenicity* in vivo

The experiment consisted of 62 RIR SPF chickens randomly assigned to two groups of twelve, and two groups of 19 for Mock and M41-K-positive control groups. At 8 days of age, chickens were inoculated via the conjunctival (eye drop) and intranasal routes with 10^4^ PFU of rIBV M41-K, M41K-N42A, M41K-G49S, or serum-free BES medium for mock infection. Clinical signs, including snicking (similar to sneezing) and rales (a vibration and/or rattle emanating from the respiratory tract) (80) were observed starting on day 1 post-inoculation (dpi). Each group of birds was observed by two to three persons over a 2 minute period, and the number of snicks during that period was recorded. The average rate of snicking per bird was then calculated. Each bird was individually assessed for the presence of rales with the total amount of rales per group recorded. On days 4 and 6 dpi, three randomly selected birds were euthanized via cervical dislocation. Ciliary activity was assessed in the harvested tracheas as described previously (13, 81). All remaining birds were culled at 7 dpi. At each sampling day, tracheal sections, eyelid, and beak tissue were stored in either RNAlater or in PBS depending on the downstream application. For the purposes of both *in vivo* studies, the eyelid includes both the upper and lower lids, and the beak refers to all soft tissue within the beak cavity including the nasal turbinates’ and nasal-associated lymphoid tissue (NALT).

#### Experiment 2: Vaccine-challenge study

One hundred 1-day-old RIR SPF chickens were randomly assigned to five groups, with each group containing 20 chicks of mixed sex. At 7 days of age, all birds were bled via a wing prick bleed. At 8 days of age, chickens were inoculated via the intraocular (eyedrop) and intranasal route with a total of 100 µL of PBS containing 10^4^ PFU of M41-R, M41R-N42A, M41K-N42A or mock infected with PBS only. Chickens were assessed for IBV-induced clinical signs from 1 day post-vaccination (dpv) to 7 dpv. The total number of snicks observed per group in a 2 minute (min) period was assessed by at least two persons. Birds were checked individually for the presence of tracheal rales. On 20 dpv, all birds were bled by wingprick and serum isolated. On 21 dpv, chickens were challenged, via the same route of inocuation as vaccination with 100 µL PBS containing 10^4^ IBV M41-CK or mock challenged with PBS only. As post-vaccination, chickens were assessed daily for the presence of IBV-induced clinical signs, from 1 day post-challenge (dpc) to 7 dpc. At 4 dpv, 4 dpc, 6 dpc, and 14 dpc, five randomly selected birds per group were culled by cervical dislocation followed by decaptiation. Blood for serum isolation was collected from exposed blood vessels in the neck. Tissues including trachea, eyelid, and beak (all soft tissue within the beak cavity including the nasal turbinates and NALT) were collected in 500 µL of PBS and 500 µL RNA later solution (ThermoFisher). On 4 dpv, 4 dpc, and 6 dpc, the tracheal ciliary activity was assessed in each trachea extracted by a method previously described (11, 13, 38); of note, the person doing the assessment was blinded to both the animal number and group.

### Virus re-isolation in chicken embryonated hen eggs

The sections of trachea, 0.02 g (experiment 1) or rings totaling a length of 1 cm (experiment 2), and eyelid tissue, accounting for one whole eyelid, both upper and lower sections, harvested as part of *in vivo* experiments 1 and 2 and stored in PBS, were homogenized in either 300 µL PBSa (experiment 1) or 500 µL (experiment 2) PBSa containing 0.005 U pencillin, 0.005 µg streptomycin (Gibco, Life Technologies), and 0.005 U nystatin (Sigma). A tissue lyser II and a 5 mm stainless steal bead were used for the homogenization. Homogenized samples were clarified by low-speed centrifugation, and 100 µL (experiment 1) or 200 µL (experiment 2) supernatant was used to infect 10-day-old RIR (experiment 1) or Valo (experiment 2) SPF chicken embryonated hen’s eggs using a 1 mL syringe and 25 G needle. Eggs were incubated for 24 h at 37°C in a tilting incubator after which they were culled via refrigeration at 4°C for at least 4 h. The allanotic fluid from each egg was harvested and RNA was extracted from 170 µL using an RNeasy mini kit (Qiagen), RNA clean-up protocol. The presence of IBV-dervied RNA was determined by RT-PCR analysis using primers specific for the 3′ UTR (79).

### Assessment of viral load by qRT-PCR

RNA from beak, eyelid, and trachea tissues harvested as part of *in vivo* experiment 1 were stored in RNA later, and RNA from infected CK cells was extracted using the RNeasy kit (QIAgen) following the manufacturer’s instructions including the on-column DNAase treatment step. For *in vivo* experiment 2 and for experimentally infected *ex vivo* TOCs, RNA was extracted from trachea and eyelid tissues using RNeasy Mini Kit (QIAgen), following the manufacturer’s instructions, including on-column DNase treatment and initial steps using TRIzol Reagent (Invitrogen) and Chloroform (Merck). Briefly, tissues were homogenized with Tissue Lyser II (Qiagen) in 1 mL TRIzol Reagent using 5 mm stainless steel beads. Samples were clarified by low-speed centrifugation and 200 µL of chloroform was added followed by a 2 min incubation at room temperature. Samples were centrifuged at 12,000 *g* for 15 min at 4°C, with the resulting upper aqueous phase subsequently mixed with 1 vol of 70% ethanol. This was then loaded to the RNeasy mini columns (Qiagen) and the remainder of the protocol was completed in accordance to the manufacturer’s instructions and included the on-column DNAse treatment step. For RNA extracted from tissues harvested as part of *in vivo* experiment 1, 500 ng RNA was transcribed using Superscript IV Reverse Transcriptase kit (Invitrogen, Waltham, MA, USA) using a random primer (5ʹ-GTTTCCCAGTCACGATCNNNNNNNNNNNNNNN-3ʹ). cDNA was normalized to 30–50 ng and quantitative real-time PCR was performed in triplicate using Taqman Fast Universal PCR Master Mix II (Life Technologies), including 125 nM final probe and 500 nM final primers. For RNA extracted from *in vivo* experiment 2, the *in vitro* and *ex vivo* experiments, the Luna Universal Probe One-Step Reaction Mix (NEB) was used with primers at a final concentration of 0.4 µM and probe at 0.2 µM and RNA at 100 ng; each sample was assessed in duplicate. Primers and probe sequences to detect levels of genomic 5′ UTR have been published previously (42, 82). Standard curves were performed to allow absolute quantitation of IBV RNA copy numbers using a plasmid containing the sequence amplified by each set of primers. The resulting Ct values were used to calculate the log of relative RNA copies (Log_10_) using the linear equation from the standard curve.

### Gene expression analysis by qRT-PCR

RNA extracted from *in vivo* derived tissues, and *in vitro* and *ex vivo* samples was investigated for gene expression. The methods of RNA extraction are detailed in the section above. A geNorm analysis was used to determine the most stable pair of reference genes for sample normalization (83, 84). The panel investigated included RPL13, HMBS, ACTB, HPRT1, B2M, and RPLPO, and results were analyzed using geNorm (85) and Normfinder (86). The most stable pair was adopted as a reference to normalize the data for the experimental genes. Primers and probes specific for the assays targeting IFNα, IFNβ, IL-6, and IL-1β were designed by Primerdesign. For tissues extracted from *in vivo* experiment 1, quantitative PCR was performed on 30–50 ng cDNA, using Taqman Fast Universal Master Mix II, no UNG (Life Technologies) including 0.3 µM final probe and 0.5 µM final primers. Samples were run in 96-well plates on the QuantStudio 5 with the default fast cycle conditions: 20 min at 95°C, 40 cycles of 1 s at 95°C, and 20 s at 60°C annealing temperature. Data were normalized using two housekeeping genes, RPLPO and ACTB. For *in vivo* experiment 2, and for *in vitro* and *ex vivo* analysis, quantitative PCR was used on 100 ng of RNA using the Luna Universal Probe One-Step Reaction Mix (NEB), with primers at a final concentration of 0.4 µM and probe at 0.2 µM. Samples were run in 96-well plates on the QuantStudio 5 with the default fast cycle conditions and an initial reverse transcriptase step: 10 min at 55°C, 20 min at 95°C, 40 cycles of 1 s at 95°C, 20 s at 60°C annealing temperature. qRT-PCR data were normalized using two housekeeping genes, RPL13 and RPLPO in tracheal organ culture and ACTB and HMBS in primary chicken kidney cells. The ∆∆CT (where CT is the cycle threshold) analysis was performed using the relative quantification of gene expression) using average ∆CT of both the reference genes in comparison with each experimental gene. Samples were run in the same plate for the reference gene and the experimental gene at the same time to avoid inter-plate variations. Data are presented as the fold change in relative mRNA gene expression of virus-infected tissue versus mock-infected tissue.

### IBV-specific antibody detection by ELISA

Serum was isolated from blood harvested during *in vivo* experiment 2 and heat-inactivated for 30 min at 56°C. Samples were diluted in sample diluent (Biocheck) either 1:80 for 4 dpv or 1:20 for 20 dpv (pre-challenge) and 4 dpc and 1:20 to 1:2560 for 14 dpc. Levels of IBV-specific antibody were determined using the IBV ELISA kit purchased from Innovative Diagnostics, ID Screen Infectious Bronchitis Indirect ELISA (IBVARSV2-10P) following the manufacturer’s protocol, with each independent test plate containing positive and negative controls. Sample:positive ratios were calculated using the following calculation [(mean sample–mean kit negative)/(mean kit positive–mean kit negative)], with those above 0.2 considered as positive.

### Quantification of IBV neutralizing antibody

Heat-inactivated serum samples harvested 14 dpc from *in vivo* experiment 2 were serially diluted in BES media from 1:2 to 1:64. Diluted serum samples were incubated for 30 min, room temperature on an orbital shaker with an equal volume of BES medium containing 10^2^ PFU of M41-CK. An untreated M41-CK sample was included in each assay. The quantity of infectious virus was determined using plaque assay in CK cells using a method previously described (69). The absence of plaques indicates the presence of neutralizing antibodies. Titers were determined using the Reed-Muench endpoint method (87) and presented as plaque reduction neutralization titer 50 values (PRNT_50_).

### Sanger sequencing

PCR products, plasmids, and primers were prepared for Sanger sequencing in accordance with the guidelines set by Source Bioscience, UK or Eurofins, Germany. All sequencing reads were either analyzed using Staden software version 2.0.0b11 or Snapgene, version 7.2. Alignments were built using CLUSTAL alignment software in UGENE version 40.1.

### Statistical analysis

All statistical analyses were performed using GraphPad Prism version 10.2. Normality and the standard deviation of each data set were assessed prior to each statistical test.

## RESULTS

### The Mac1 domain of coronaviruses that infect mammalian and avian host species demonstrates a high degree of conservation of specific residues

The sequence of the IBV Mac1 domain was derived from reference 62, which described the crystal structure of IBV M41, which accounts for the numbering of nucleotide residues 3523 to 4002 of the M41-CK reference sequence (accession number MK728875). Of note, this sequence is two amino acids longer at the amino-terminal end and 14 amino acids shorter at the carboxy-terminal end than the sequence used by Piotrowski et al. (63), who published the crystal structure of the Mac1 domain from the Beaudette strain (63). This sequence is also different from that published by Fehr et al. (58), whose sequence of the IBV Mac1 domain is three amino acids shorter at the amino-terminal end than that used by Xu et al. (62). For clarity and due to the discrepancies on the exact length of the Mac1 domain sequence, in this study, we have used the sequence of the Mac1 domain as published by Xu et al. (62) as the reference sequence. This is the longest sequence and as it was used to generate the crystal structure of the M41 Mac1 domain, it is more directly related to this study. In addition, when introducing the Mac1 domain sequences from other avian coronaviruses, we aligned the equivalent amino acid residues to the M41 reference sequence, indicated above, to keep the amino acid lengths of the various Mac1 domains the same.

**Fig 1:**
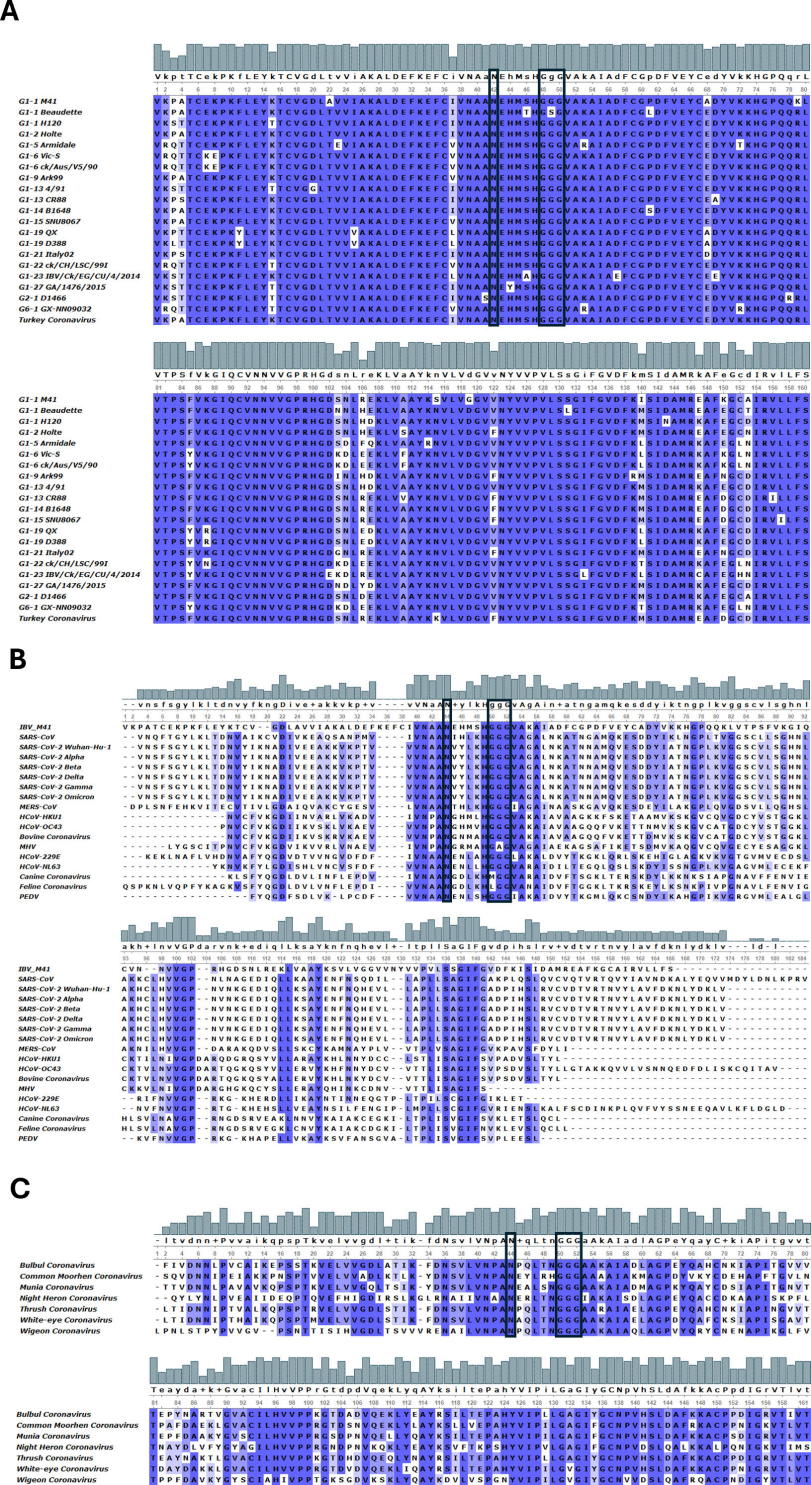
Residues within the Mac1 domain are conserved between coronaviruses that infect mammalian and avian species. Highlighted by the black boxes are the triple Glycine motif and the highly conserved Asn residue. (**A**) IBV strains: Beaudette (AJ311317), H120 (ON350836), M41 (MK937830), Gray (GU393334.1), Armidale (KU556805), Vic-S (KF460437.1), ckAusV590 (MK990812), ck/ch/LHLJ/110664 (MN509589), Ark99 (MH779860.1), 4/91 (KF377577.1), CR88 (MN548285), B1648 (KR231009), D388 (MN54828971), QX (MN548289), Italy02 (MN548288), IBV/Ck/EG/CU/4/2014 (KY805846), GA/1476/2015 (MN599049), D1466 (MN548286), GX-NN09032 (JX8979001), and the Turkey Coronavirus (NC010800.1) (**B**) Alpha- and betacoronaviruses: MERS-CoV (MK967708.1), SARS-CoV (AY278488.2), SARS-CoV-2 Wuhan-Hu-1 (NC045512.2), SARS-CoV-2 Alpha (OV054768.1), SARS-CoV-2 Beta (OX008586.1), SARS-CoV-2 Delta (OK091006.1), SARS-CoV-2 Gamma (OR578388.1), SARS-CoV-2 Omicron (OL672836.1), HCoV-229E (PP810610), HCoV-NL63 (JX504050.1), HCoV-HKU1 (KF686346.1), HCoV-OC43 (KX344031.1), Bovine coronavirus (LC830477), Canine coronavirus (PP526172.1), Feline Coronavirus (KX722531), MHV (AY700211), PEDV (MK841495.1). (**C**) Deltacoronaviruses: Wigeon coronavirus HKU20 (NC016995), White-eye coronavirus HKU16 (NC_016991.1), Thrush coronavirus HKU12 (NC011549.1), Night heron coronavirus HKU19 (NC016994.1), Munia coronavirus HKU13 (NC_011550.1), Common moorhen coronavirus HKU21 (NC016996.1), and Bulbul coronavirus HKU11 (NC_011547.1).

An alignment of the Mac1 domain sequences from a diverse panel of IBV strains representing several genotypes and serotypes as well as the *Gammacoronavirus* Turkey Coronavirus demonstrated a high degree of amino acid conservation, with the previously researched Asn at residue 42 within the catalytic core of the Mac1 domain (58) completely conserved (Fig. 1A). This is in line with other coronaviruses including MHV, SARS-CoV, and SARS-CoV 2 (Fig. 1B) and the avian deltacoronaviruses (Fig. 1C). The triple glycine motif, Gly-Gly-Gly which the crystal structures have suggested lines the ADRP-binding cleft (62, 63), at residues 48–50 is also conserved amongst IBV strains with the exception of the attenuated IBV strain Beaudette which exhibits Gly-Ser-Gly (Fig. 1A). Unlike the conserved Asn residue, the triple glycine motif does present variably between coronaviruses, with strains of Feline Coronavirus containing either Val-Gly-Gly, Met-Gly-Gly or Leu-Gly-Gly (accession numbers: JN183883.1, FJ938053.1, FJ938051.1, JN634064.1, FJ938060.1, and GQ152141.1), Canine Coronavirus containing Met-Gly-Gly (accession numbers: KP849472.1, JQ404409.1, GQ477367.1, JQ404410.1, KP981644.1, KC175340.1, KC175341.1, KY063616.1, MZ420153.1, PP526172.1, JN856008.2, OM950728.1, and OM950729.1) and MHV containing Gly-Ala-Gly (NC_048217.1.1, AC_000192.1, FJ647219.1, OK491634.1, FJ647223.1, AF201929.1, FJ647224.1, AF208067.1, AF208066.1, and JQ173883.1). Investigation of the sequence of avian deltacoronaviruses shows that these do, like IBV, contain the triple Glycine motif (Fig. 1C).

### Generation of the rIBVs containing mutations within the macrodomain

Guided by previous research investigating the conserved Asn residue (57, 58, 60, 64, 65) and the IBV crystal structures (62, 63) as well as by these alignments two rIBVs were generated using a vaccinia virus-based reverse genetic system previously established on the pathogenic IBV M41-CK, a Massachusetts serotype, GI-1 genotype (41). The first rIBV, M41K-N42A, contains two nucleotide mutations, AA_3646_-_3647_GC, within the catalytic core of the Mac1 domain resulting in the amino acid change Asp to Ala at residue 42 (N42A); this mutation is predicted to reduce the catalytic activity of the Mac1 domain (58) (57, 65). The second rIBV M41K-G49S, contains the single point mutation G_3667_A, resulting in the amino acid change, Gly to Ser at residue 49 (G49S). This mutation altered the conserved triple glycine motif lining the binding cleft, placing a serine in the central position (Gly-Ser-Gly), therefore potentially altering the structure to that exhibited by the attenuated Beaudette strain. It is hypothesized that this change of structure may affect the binding of the ADP-ribose substrate and therefore Mac1 activity (45, 63). Each rIBV was rescued in primary CK cells, passaged three or four times to increase viral titers, and propagated once in embryonated eggs to generate the viral stock. Both rIBV M41K-G49S and M41K-N42A were successfully rescued. The presence of the relevant mutations alongside the remaining Nsp3 sequence in each rIBV was confirmed to be as expected. Subsequent full-genome sequencing confirmed that the genomic sequence of M41K-N42A was as expected with no additional mutations identified. Unexpectedly, the M41K-G49S contained two additional mutations, T20455C resulting in a Phenylalanine to Leucine amino acid change at residue 34 in S and T24213G resulting in a Serine to Alanine mutation at residue 7 in E.

### Both the N42A and G49S mutations were stably maintained during serial passage

Both M41-K-N42A and M41-K-G49S were passaged a total of ten times in embryonated SPF hens’ eggs and CK cells alongside the parental M41-K. At passage 10, the sequence of the Mac1 was analyzed by Sanger sequencing; no changes were identified. Both the mutations N42A and G49S were therefore stably maintained for at least 10 passages *in vitro* and *in ovo*.

### M41K-N42A exhibited reduced replication *in ovo* and *ex vivo*

**Fig 2:**
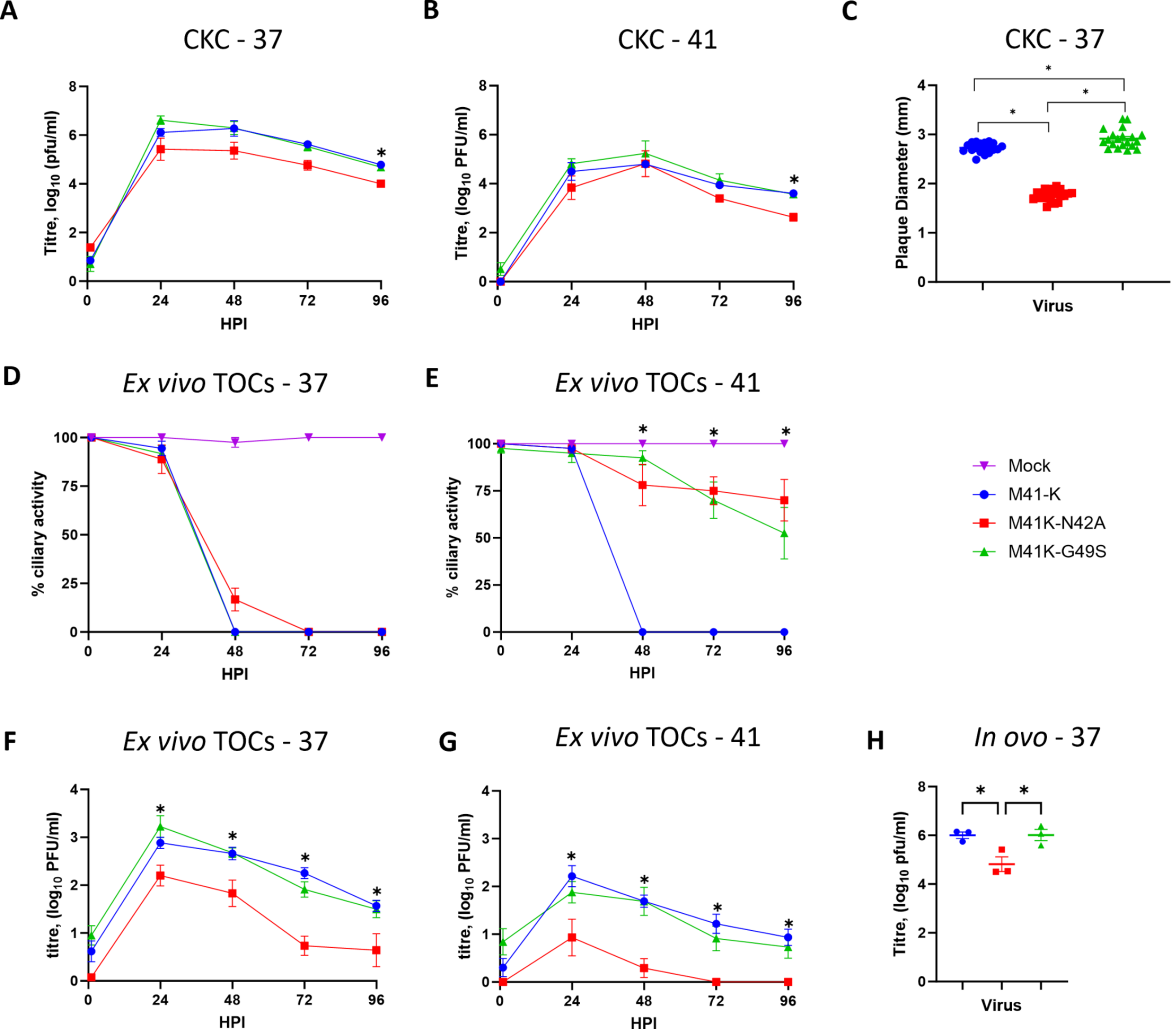
M41K-N42A exhibits reduced replication *ex vivo* and *in ovo*. Primary CK cells (**A–C**), *ex vivo* tracheal organ cultures (TOCs; **D–G**) and 10-day old SPF embryonated hen’s eggs (**H**) were inoculated with 10^4^ PFU (**A, B, D–G**), 20^3^ PFU (**H**) or serially diluted MOI (0.1–0.001; (**C**) of either M41-K, M41K-N42A, M41K-G49S or media only for mock infection, and either incubated at 37 (**A, C, D, F, H**) or 41°C (**B, E, G**). The supernatant (**A, B, F, G**) was harvested at 24 h intervals and allantoic fluid (**H**) at 24 hpi with the quantity of infectious virus determined via plaque assay in CK cells. (**D and E**) The ciliary activity of each TOC was measured using a light microscope at 24 h intervals and a mean of 10 technical replicates per experiment was calculated. (**C**) The diameter of 20 plaques was measured using image J after staining with 0.1% crystal violet 4 days post-infection. (**A–H**) Error bars represent the standard error of the mean of three independent experiments, with statistical differences, highlighted by * (*P* < 0.05) were assessed using a two-way ANOVA (**A, B, D–G**) or a one-way ANOVA (**C, H**) with Tukey test for post hoc comparisons. (**A, B**) Titers of M41K-N42A are reduced in comparison to M41K-G49S and M41-K at 96 hpi. (**D,E**) From 48 hpi, all CA is reduced in infected groups compared to mock; not highlighted. Highlighted differences are between M41-K and both M41K-N42A and M41K-G49S (**F, G**) Statistical differences highlight reduced titers of M41K-N42A compared to both M41-K and M41K-G49S.

Where appropriate i*n vitro* growth kinetics were investigated at both 37 and 41°C, as temperature has been demonstrated to impact replication, with a decrease in replication, at the higher temperature being previously associated with an attenuated *in vivo* phenotype (42, 67, 88). The chicken respiratory tract exhibits a temperature gradient with the lower respiratory tract akin to core body temperature, 41°C and the upper respiratory tract more akin to 37°C (67, 89). In primary CK cells, the growth kinetics of both M41K-N42A and M41K-G49S were comparable to each other and to parental M41-K (Fig. 2A and B). Although titers of M41K-N42A appear lower than both M41-K and M41K-G49S, statistical significance is only reached at 96 hours post-infection (HPI). The plaque size was reduced following infection with M41K-N42A when compared to infection with M41K-G49S and M41-K (Fig. 2C). During *in vivo* infection, IBV reduces ciliary activity within the trachea and this is routinely used as a marker for IBV replication (13). Tracheal ciliary activity reduces as IBV replicates (13, 38, 90). In *ex vivo* tracheal organ cultures (TOCs) ciliary activity was rapidly reduced and lost following infection with any of the three viruses at 37°C (Fig. 2D). Interestingly, total loss of ciliary activity occurred 24 h later for M41K-N42A in comparison to TOCs infected with M41K-G49S or M41-K (Fig. 2D). During infection of TOCs with either M41K-N42A or M41K-G49S the associated ciliary activities were retained to a greater extent in comparison to infection with parental M41-K at 41°C (Fig. 2E), although M41K-G49S caused greater loss than M41K-N42A. This observed retention of ciliary activity observed in TOCs infected with M41K-N42A or M41K-G49S was further investigated. A decrease in replication, as indicated by the production of infectious progeny, was observed at both 37°C and 41°C for M41K-N42A in comparison to both M41-K and M41K-G49S (Fig. 2F and G), with a more marked decrease at 41°C (Fig. 2G). Despite the retention of ciliary activity of M41K-G49S at 41°C (Fig. 2E), a decrease in replication was not observed (Fig. 2G). As IBV vaccine viruses are produced in embryonated hens’ eggs, the quantity of infectious progeny during *in ovo* infection was also investigated. A decrease in infectious progeny was only observed *in ovo* for M41K-N42A (Fig. 2F). While neither of the mutations to the Mac1 domain has impacted *in vitro* replication in primary CK cells, the N42A mutation has negatively affected replication in both *ex vivo* TOCs and *in ovo*.

### M41K-N42A is attenuated *in vivo*

**Fig 3:**
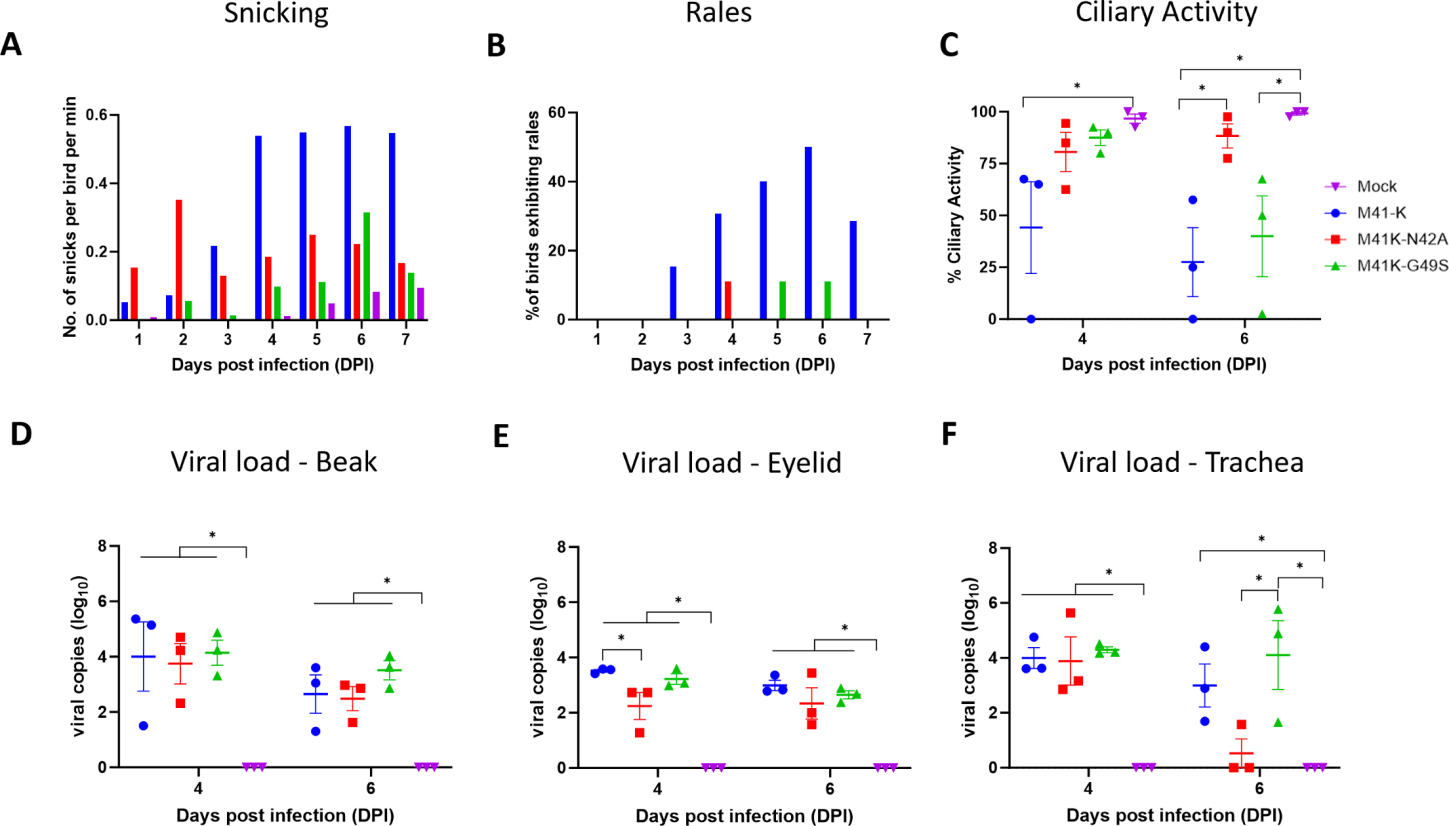
M41K-N42A exhibits reduced pathogenicity *in vivo*. Groups of 12 8-day-old chicks were inoculated via the intranasal and intraocular route with 10^4^ PFU of M41-K, M41K-N42A, M41K-G49S, or mock infected with PBS. (**A**) Post-infection, the number of snicks per group was independently counted by at least two persons with the mean average calculated at the number of snicks per bird per min. (**B**) Chickens were individually assessed for the presence of tracheal rales, with a percentage positive for rales in each group calculated. (**C**) At 4 and 6 dpi, the trachea was extracted from three randomly chosen birds per group, and ten 1 mm rings were sectioned and the ciliary activity in each ring was scored. The average (mean) activity of the rings derived from each bird was calculated. (D–F) RNA was extracted from beak, eyelid, and tracheal tissue from the same birds and assessed by RT-qPCR for the quantity of IBV-derived RNA using primers and probes specific for the 5′ UTR. (**C–F**) Each plotted point represents a single bird with error bars representing SEM. Statistical differences highlighted by * (*P* < 0.05) were assessed using a two-way ANOVA with a Tukey test for post hoc analysis.

Groups of 8-day-old SPF chickens were inoculated via the intranasal and intraocular route with M41K-N42A, M41K-G49S, the pathogenic parental virus M41-K or mock infected with serum-free media. Chickens were assessed daily for the presence of clinical signs associated with IB including snicking (Fig. 3A) and tracheal rales (Fig. 3B). Chickens infected with M41K-N42A exhibited a higher number of snicks than all other groups at 1 and 2 dpi. On all other days, snicking was less in comparison to the M41-K pathogenic control group. M41K-G49S infected chickens exhibited less snicking on all days in comparison to the M41-K group and from all days except 6 dpi when compared to M41K-N42A. The percentage of chickens exhibiting rales was less for both M41K-N42A- and M41K-G49S-infected groups in comparison to the M41-K-infected group, with only sporadic rales observed between 4 and 6 dpi. As expected, mock-infected chickens exhibited minimal levels of snicking and no rales.

Tracheal ciliary activity is used to measure IBV pathogenicity (11, 13) and as such was assessed from three randomly selected chickens on 4 and 6 dpi. At 4 dpi, tracheal ciliary activities were comparable in chickens infected with M41K-N42A and M41K-G49S, with mean average ciliary activities of 80.6% and 87.5%, respectively; this was comparable to the mock-infected group at 97%. Ciliary activities in birds infected with M41-K were 44% in comparison (Fig. 3C). Similar to the observations at 4 dpi, the ciliary activities for tracheas extracted at 6 dpi from the M41K-N42A infected chickens were comparable to those extracted from the mock-infected group, average (mean) of 88.3% and 99.2%, respectively (Fig. 3C). The observed tracheal ciliary activities from the M41K-G49S group, however, were lower than both the mock-infected and M41K-N42A groups and more comparable to those observed from M41-K group (Fig. 3C). This indicates that M41K-G49S demonstrated an *in vivo* phenotype more comparable to the pathogenic parent virus M41-K at 6 dpi. More interestingly, our results indicated that M41K-N42A exhibited an *in vivo* phenotype that could be considered attenuated.

### M41K-N42A exhibited reduced viral load in tracheal tissue at 6 dpi

Tissues representing the sites of M41 infection were harvested on both 4 and 6 dpi and RNA was extracted. This included beak tissue composed of nasal connective tissue and lymphoid tissue, eyelid, and trachea. The quantities of IBV-derived RNA in these tissues were assessed (Fig. 3D through F) and found to be largely comparable between the two rIBVs and the pathogenic control M41-K from the beak tissues on both 4 and 6 dpi (Fig. 3D) and from tracheal tissues on 4 dpi (Fig. 3F). Differences were observed on 4 dpi in the eyelid (Fig. 3E) and 6 dpi in the tracheal tissues (Fig. 3F), in which M41K-N42A exhibited a lower relative copy number in comparison to the parental M41-K.

As the trachea is considered the main site of IBV replication, tracheal tissue was homogenized to generate a tissue-derived supernatant that was used to inoculate embryonated hen’s eggs to determine if the infectious virus was present. While infectious virus was recovered from trachea harvested from M41-K and M41K-G49S birds on both 4 and 6 dpi, it could only be recovered from M41K-N42A-infected birds on 4 dpi (Table 1). This alongside the absence of IBV-derived RNA in two of three birds (Fig. 3F) suggests that M41K-N42A has been cleared from the trachea at 6 dpi.

**TABLE 1:** Presence of infectious IBV in tracheal tissue^*a*^

Group	4 dpi	6 dpi
Mock	0/3	0/3
M41K-N42A	3/3	0/3
M41-G49S	3/3	3/3
M41-K	3/3	2/3

^*a*^ Virus presence was determined from randomly selected birds with results displayed as the number of positive birds/total number of birds sampled. Eyelid and tracheal tissues were homogenized in PBSa to generate a tissue-derived supernatant that was used to infect embryonated hens’ eggs for potential virus growth. Allantoic fluid derived from the infected eggs was assessed, by RT-PCR, for the presence of IBV-derived RNA indicative of IBV growth.

### The effect of the Mac1 domain mutations on host responses may be dependent on tissue type

**Fig 4:**
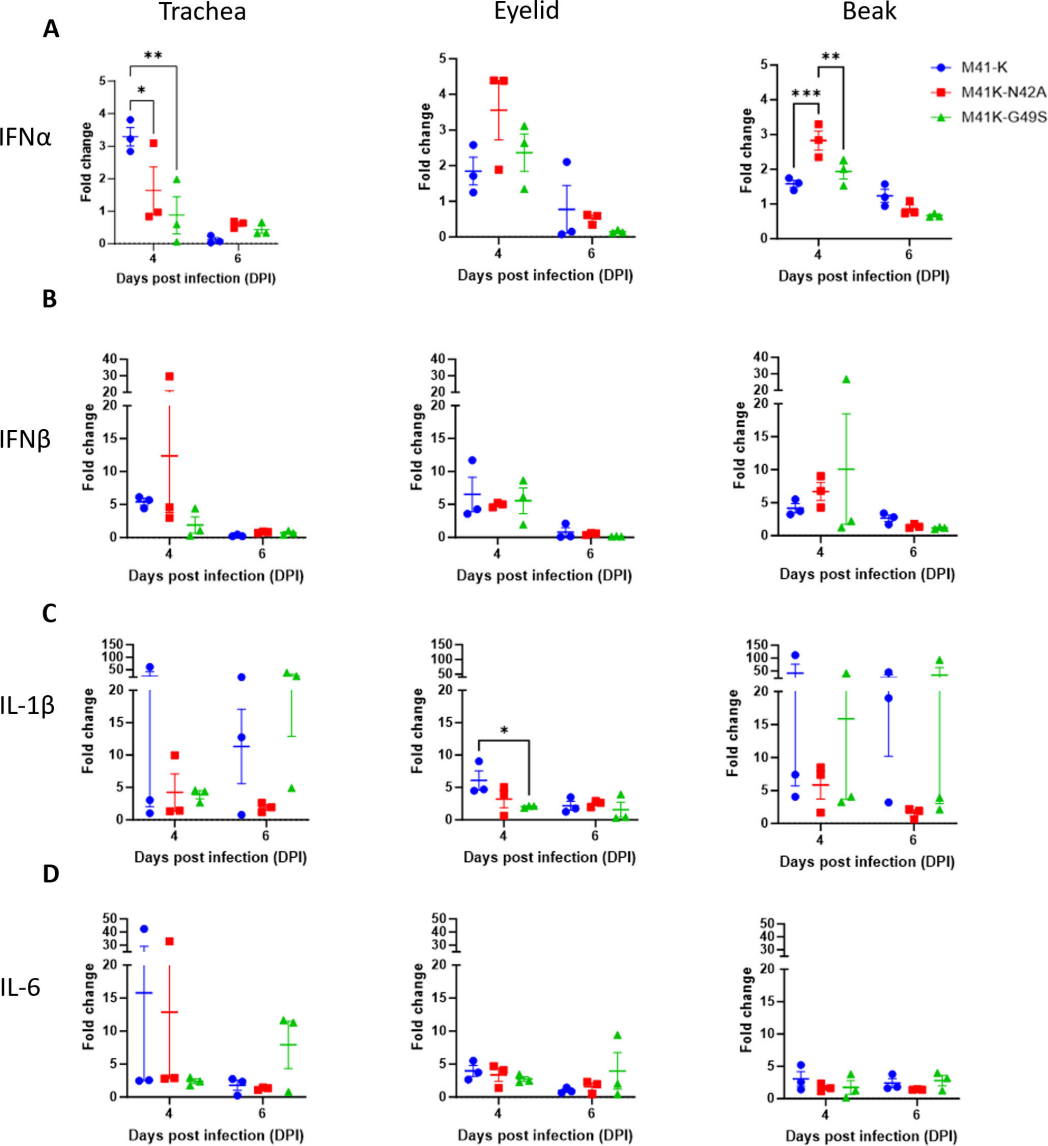
The effect of the Mac1 domain mutations on host responses varies between tissue types. RNA extracted from tracheal, eyelid, and beak tissue were analyzed by qRT-PCR for IFN-α (**A**), IFNβ (**B**), IL-1β (**C**), and IL-6 (**D**). All data were normalized to RPLPO and ACTB, and fold changes were calculated compared to mock-infected tissue. Statistical differences highlighted by * (*P* < 0.05) were analyzed using a two-way ANOVA with a Tukey test for post hoc analysis. Each plotted point represents an individual bird and error bars represent SEM.

The coronavirus Mac1 domain has been implicated in the regulation of inflammatory cytokine and interferon (IFN) responses during viral infection (61, 91). Total RNA extracted from beak, eyelid, and tracheal tissues harvested on days 4 and 6 pi was investigated for the expression profiles of the IFNα, IFNβ, IL-1b, and IL-6 genes. The IFN genes were targeted as they have been implicated in host responses from research into Mac1 mutants of MHV, HCoV 229E, SARS-CoV, and SARS-CoV 2 (58, 59, 61, 92), and the cytokines IL-1b and IL-6 have been implicated during IBV infection (93, 94). Following infection with the rIBVs M41K-N42A and M41K-G49S, only differences in IFNα expression were observed (Fig. 4). A decrease in the expression of IFNα was observed in tracheal tissue examined from birds infected with either M41K-N42A or M41K-G49S at 4 dpi but with an increased expression observed in beak tissue (for M41K-N42A only) when compared to those infected with M41-K (Fig. 4A). No difference in IFNβ expression was observed in any tissue investigated (Fig. 4B). Analysis of the expression of the cytokines, IL-1β and IL-6, indicated that birds infected with M41K-G49S at 4 dpi exhibited a decrease in IL-1β expression in eyelid tissue only (Fig. 4C and D).

**Fig 5:**
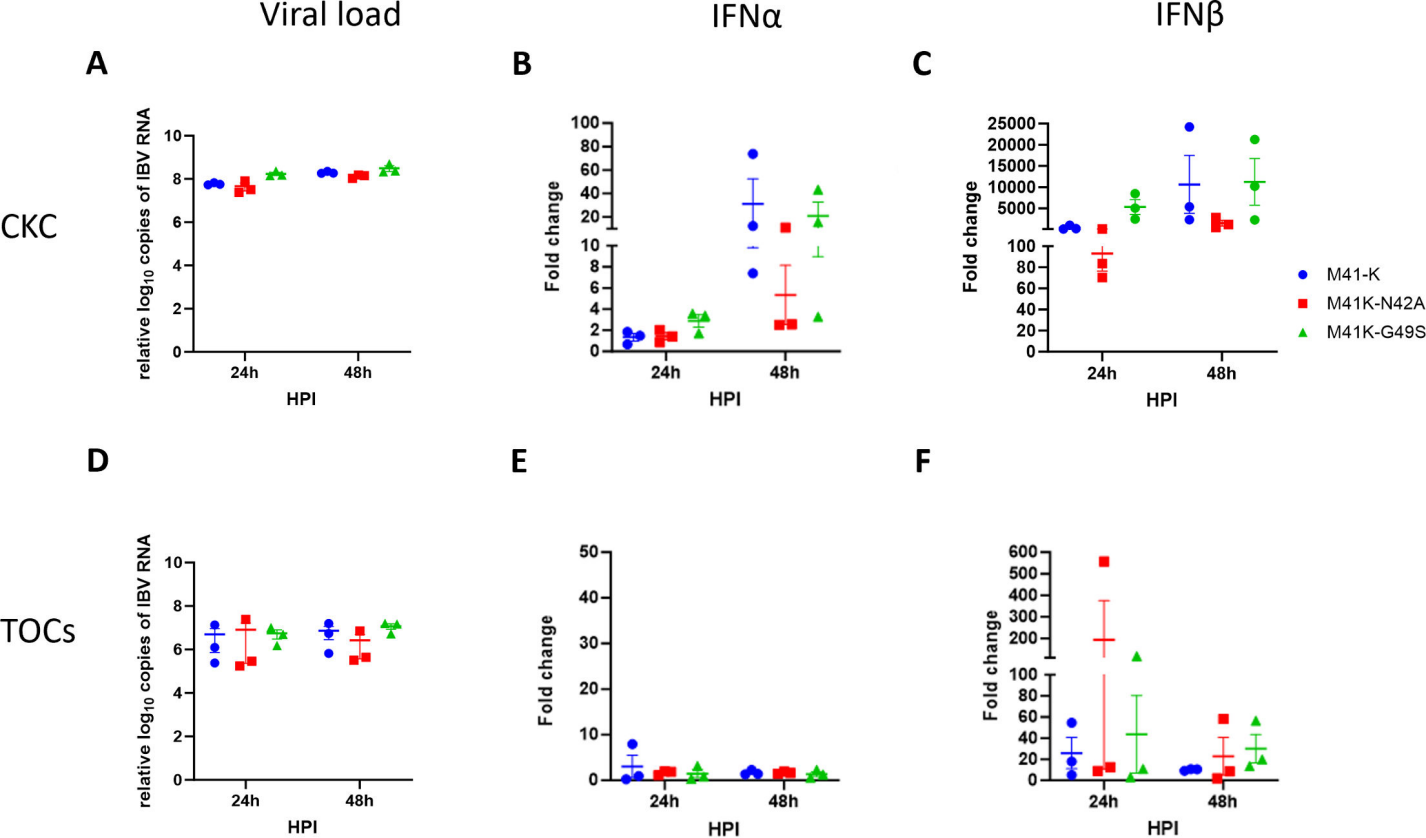
Mutations to the Mac1 domain have not affected IFNα or IFNβ response *in vitro* nor *ex vivo*. Primary CK cells (**A–C**) or *ex vivo* tracheal organ cultures (TOCs; **D–F**) were infected with 10^4^ PFU M41-K, M41K-N42A, M41K-G49S, or mock infected with media only. RNA extracted at 24 and 48 hpi was analyzed by qRT-PCR for the quantity of IBV-derived RNA (**A, D**), IFNα (**B, E**), IFNβ (**C, F**). Data were normalized to RPL13 and RPLPO in tracheal organ culture and ACTB and HMBS in primary chicken kidney cells, and fold changes were calculated compared to mock. Statistical differences were analyzed using a two-way ANOVA with a Tukey test for post hoc analysis; no differences were identified. Each plotted point represents an individual bird and error bars dictating SEM.

To investigate further, primary CK cells and *ex vivo* TOCs were infected with either M41-K, M41K-N42A, M41K-G49S or mock infected (Fig. 5). Equivalent quantities of IBV-derived RNA were detected suggesting comparable replication (Fig. 5A and D). While it appears that at 48 hpi, M41K-N42A infected is associated with a reduction in IFNα expression in comparison to both M41-K and M41K-G49S, statistical significance was not reached. Similarly, no statistical differences in IFN β expression were detected at either 24 or 48 hpi in comparison to the parental M41-K.

### Generation of rIBVs containing attenuating mutations in Mac1, Nsp10, and Nsp14

As the results of our *in vivo* study highlighted that the N42A mutation proved to be more attenuating in terms of retention of tracheal ciliary activity (Fig. 3C), we engineered the N42A mutation into the genome of M41-R, an attenuated rIBV (41). M41-R contains four individual point mutations in Nsp10, 14, 15, and 16 resulting in the amino acid changes, Pro85Leu, Val393Leu, Leu183Ile, and Val209Ile, respectively (41). While the M41-R backbone contains all four mutations, our previous research demonstrated that it was the amino acid changes in Nsp10 and 14 that are associated with both the attenuated *in vivo* phenotype and a temperature-sensitive replication phenotype, in which replication is reduced at 41°C, the core body temperature of a chicken (41, 42). The resulting rIBV denoted M41R-N42A was recovered in CK cells with a stock generated at passage 4; the presence of the modifications was confirmed by Sanger sequencing. Subsequent full-genome analysis identified two additional mutations in S, A20635T and C22924T resulting in the amino acid changes Serine to Cysteine and Leucine to Phenylalanine at residues 94 and 857, respectively. In addition, two nucleotides, GT at position 25181–2 were deleted in the putative accessory gene 4b.

**Fig 6:**
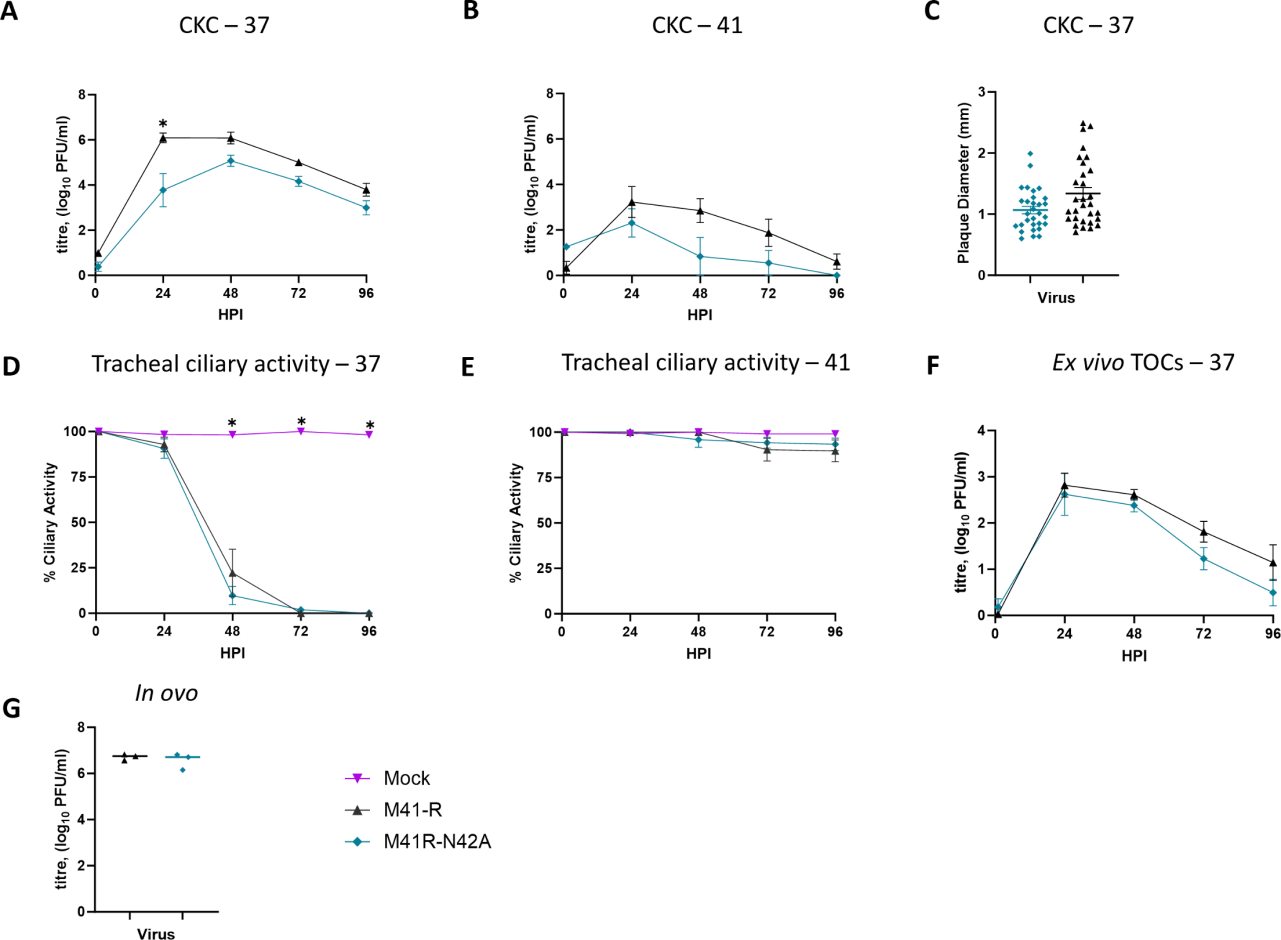
M41R-N42A replicates comparably to M41-R *in vitro*, *ex vivo,* and *in ovo*. The N42A mutation was incorporated into M41-R, a virus which is attenuated *in vivo* and that contains four mutations, one in each of Nsp10 (Pro85Leu), 14 (Val393Leu), 15 (Leu183Ile), and 16 (Val209Ile) (41). Primary CK cells (**A–C**) and *ex vivo* tracheal organ cultures (TOCs; **D–F**) and 10-day-old SPF embryonated hen’s eggs (**G**) were inoculated with 10^4^ PFU (**A, B, D–F**), 20^3^ PFU (**G**) or MOI 0.01–MOI 0.001 (**C**) with either M41-R, M41R-N42A or media only for mock infection, and either incubated at 37 (**A, C, D, F, G**) or 41°C (B, E). The supernatant (**A, B, F**) was harvested at 24 h intervals and allantoic fluid (**G**) at 24 hpi with the quantity of infectious virus determined via plaque assay in CK cells. (**D and E**) The ciliary activity of each TOC was measured using a light microscope at 24 h intervals and a mean of 10 technical replicates per experiment was calculated. (**C**) The diameter of 20 plaques was measured using image J after staining with 0.1% crystal violet 4 days post-infection. Error bars represent the SEM of three independent experiments, with statistical differences, highlighted by * (*P* < 0.05), assessed using a two-way ANOVA (**A, B, D, E**) or an unpaired T-test (**C, G**) with Tukey test for post hoc comparisons.

Growth analysis at both 37°C and 41°C demonstrated that rIBV M41R-N42A exhibited reduced replication compared to the parental M41-R at 24 hpi at 37°C in CK cells (Fig. 6A). Plaque size, replication in *ex vivo* TOCs as indicated by both tracheal ciliary activity and infectious progeny, as well as replication *in ovo* were comparable (Fig. 6B through F). In addition, serial passage *in ovo* and CK cells demonstrated that the mutations in the Mac1 domain as well as the attenuating mutations in Nsp10 and Nsp14 were stably maintained for at least 10 passages.

### Vaccination of chickens with rIBVs containing the N42A mutation protects against homologous challenge

**Fig 7:**
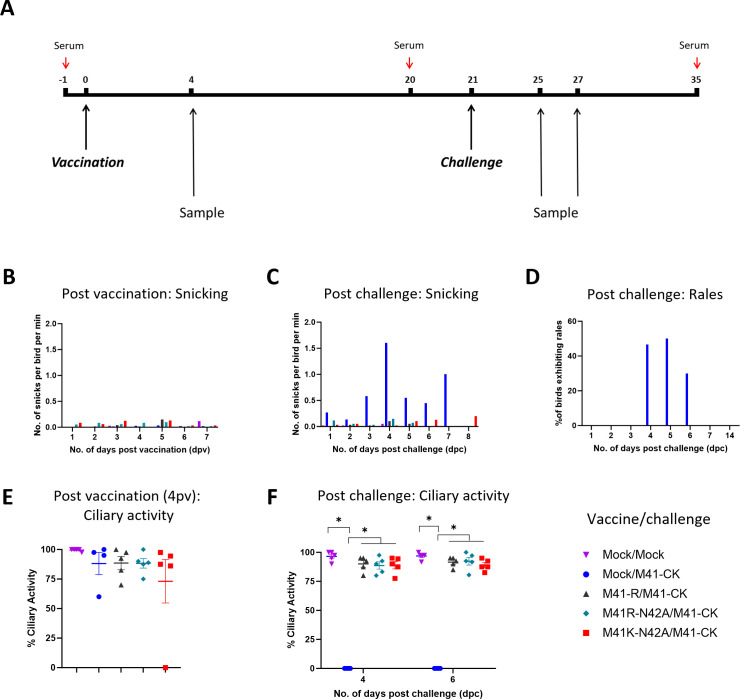
Vaccination with M41K-N42A or M41R-N42A protects against homologous challenge. (**A**) Schematic detailing protocol for *in vivo* vaccine challenge experiment. Chickens were randomly assigned to one of five groups of 20 and were vaccinated with 10^4^ PFU of M41-R, M41K-N42A, M41R-N42A, or mock infected with PBS at 7 days of age via the intraocular and intranasal route. Via the same intraocular and intranasal route, at 21 dpv, the birds were challenged with either 10^4^ PFU of M41-CK or mock challenged with PBS. Chickens were observed daily for the presence of snicking (**B, C**) and tracheal rales (D). The number of snicks per group was independently counted by at least two persons with the mean average calculated as the number of snicks per bird per min. Chickens were individually assessed for the presence of tracheal rales, with percentages positive for rales in each group calculated. No rales were observed post-vaccination. At 4 dpv (**E**) and 4 and 6 dpc (**F**), five birds were culled per group and the trachea was harvested. Per trachea ten 1 mm rings were sectioned and the ciliary activity of each ring was scored with the average (mean) activity of the rings calculated. Each plotted point represents an individual bird with error bars representing SEM. Statistical differences (*P < 0.05) were assessed using a two-way ANOVA with a Tukey test for post hoc analysis. The experiment ended at 14 dpc with all remaining birds culled.

Groups of 20 chickens were vaccinated via the intraocular and intranasal route with M41K-N42A, M41R-N42A, M41-R, or mock vaccinated with PBS and challenged with M41-CK or mock challenge with PBS 21 days later as outlined in Fig. 7A. Clinical signs including snicking (Fig. 7B) and rales were observed post-vaccination and as expected no IB-associated clinical signs including tracheal rales were observed. Tracheal ciliary activity was observed in tracheas extracted from five randomly selected chickens per group on day four post-vaccination (dpv); activities were comparable between the M41-R and M41R-N42A and mock-vaccinated groups (Fig. 7E), although one bird vaccinated with M41K-N42A exhibited a complete loss of ciliary activity (Fig. 7E). Twenty-one dpv birds were challenged, via the same route as vaccination, with either PBS for mock challenge or with virulent IBV M41-CK. Clinical signs were assessed, with only chickens in the mock vaccinated/M41-CK challenged group (Mock/M41-CK) exhibiting snicking and tracheal rales (Fig. 7C and D). In line with the requirements set by the European Pharmacopeia (95) tracheal ciliary activities were observed in five randomly selected birds at both 4- and 6-days post-challenge (dpc; Fig. 7F). As expected on both 4 and 6 dpc, ciliary activities were retained in mock vaccinated/mock challenged birds (Mock/Mock) and the M41-R-vaccinated/M41-CK-challenged birds (M41-R/M41-CK) and were abolished in the Mock/M41-CK group. Both the rIBVs M41K-N42A and M41R-N42A-vaccinated/M41-CK-challenged groups, M41K-N42A/M41-CK and M41R-N42A, respectively, exhibited retained tracheal ciliary activities, comparable to both the Mock/Mock and M41-R/M41-CK groups. For a bird to be considered fully protected, the European Pharmacopeia states that at least 50% ciliary activity must be retained post-challenge in 9 of the 10 tracheal rings assessed (95); all sampled tracheas on both 4 and 6 dpc from the M41-R, M41K-N42A, and M41R-N42A-vaccinated groups met this standard. By measuring both clinical signs and tracheal ciliary activities, vaccination with the rIBVs M41K-N42A and M41R-N42A could therefore induce a fully protective response against a virulent M41-CK challenge.

### Challenge virus could not be detected in M41K-N42A- nor M41R-N42A-vaccinated chickens

**Fig 8:**
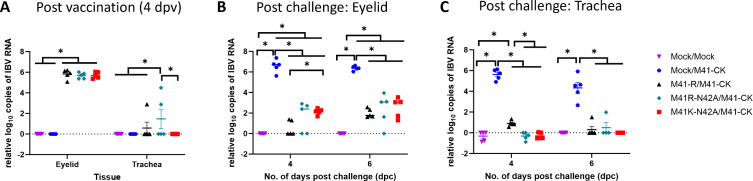
Vaccination with M41K-N42A or M41R-N42A reduces viral replication post-challenge. RNA extracted 4 dpv (**A**) and 4 and 6 dpc (**B, C**) were assessed for the quantity of IBV-derived genomic RNA by quantitative RT-PCR using primers and probes specific for the 5′ UTR. Each plotted point represents an individual animal with error bars indicating SEM. Statistical differences, highlighted by *P < 0.05, were evaluated using a (**A**) one- or (**B, C**) two-way ANOVA with a Tukey test for post hoc analysis.

Eyelid and trachea tissues were harvested both post-vaccination and post-challenge and RNA was extracted. This RNA was analyzed by qRT-PCR for the presence of IBV-derived RNA (Fig. 8). In eyelid tissue, IBV-derived RNA was detected in all rIBV-vaccinated birds post-vaccination but in tracheal tissue IBV-derived RNA was only detected in three birds, one vaccinated with M41-R and two vaccinated with M41R-N42A (Fig. 8A). No infectious virus was re-isolated from tracheal tissue post-vaccination (Table 2). Post-challenge either very low levels of IBV-derived RNA or no RNA could be detected in the rIBV-vaccinated groups (Fig. 8B and C). In all cases, the quantity of RNA detected was significantly lower than in the Mock/M41-CK control group. Of note, no IBV-derived RNA was detected in the tracheas of the M41R-N42A or M41K-N42A post-challenge, with only minimal levels detected in those vaccinated with M41-R (Fig. 8). No infectious virus could be isolated from either eyelid or tracheal tissue harvested post-challenge in any of the rIBV-vaccinated groups (Table 2).

**TABLE 2:** Presence of infectious virus in tissues harvested from vaccine-challenge experiment^*a*^

Group	4 dpv		4 dpc		6 dpc		14 dpc	
	Eyelid	Trachea	Eyelid	Trachea	Eyelid	Trachea	Eyelid	Trachea
Mock/Mock	0/5	0/5	0/5	0/5	0/5	0/5	0/5	0/5
Mock/M41-CK	0/5	0/5	4/5	5/5	3/5	2/5	0/5	0/5
M41-R/M41-CK	1/5	0/5	0/5	0/5	0/5	0/5	0/5	0/5
M41K-N42A/M41-CK	4/5	0/5	0/5	0/5	0/5	0/5	0/5	0/5
M41R-N42A/M41-CK	4/5	0/5	0/5	0/5	0/5	0/5	0/5	0/5

^*a*^ Virus presence was determined from randomly selected birds at 4 dpv and 4, 6 and 14 dpc with results displayed as the number of positive birds/total number of birds sampled. Harvested eyelid and tracheal tissue were homogenized in PBSa to generate a tissue-derived supernatant that was used to infect embryonated hens’ eggs for viral re-isolation. The resulting allantoic fluid was assessed by RT-PCR for the presence of IBV-derived RNA indicative of infectious progeny.

### Vaccination with Mac1 domain mutants induced a robust antibody response

**Fig 9:**
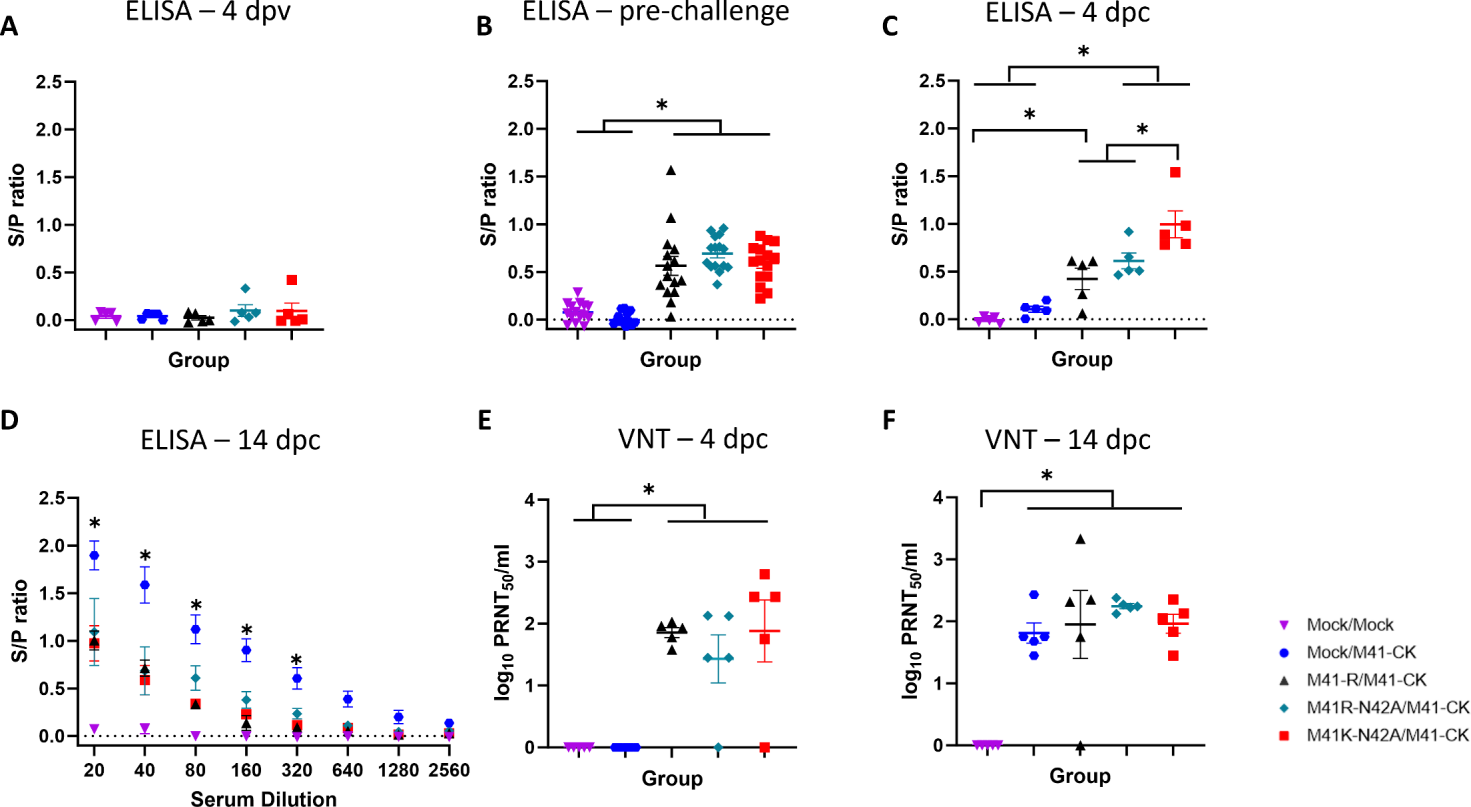
Vaccination with either M41K-N42A or M41R-N42A induces a robust neutralizing response. Serum was isolated from blood collected post-cull from five randomly chosen chickens on (**A**) 4 dpv and 4 (**C, E**) and 14 dpc (**D, F**) and (**B**) from all birds on 20 dpv, which was 1 day prior to challenge. Serum was diluted (**A**) 1:80, (**B, C**) 1:20 and (**D**) 1:20 to 1: 2,560 for ELISA using the commercial Innovative Diagnostics ID Screen IBV ELISA kit. Samples were run in triplicate, and the average (mean) S/P ratio was calculated. S/P ratios are displayed with error bars representing the SEM of individual animals. To assess the levels of neutralizing antibody, serum from (**E**) 4 and (**F**) 14 dpc was serially diluted and incubated with 100 PFU of M41-CK, followed by a plaque assay on CK cells to determine the PRNT_50_. The PRNT_50_ values for each bird are displayed, with error bars representing SEM. Statistical differences were assessed by (A–C, **E, F**) one-way ANOVA or a (**D**) a two-way ANOVA followed by a Tukey test for multiple comparisons.

Blood was harvested at specific time points pre-vaccination, post-vaccination, and post-challenge (Fig. 7) and serum was isolated, which was assessed for the presence of IBV-specific antibodies by enzyme-linked immunosorbent assay (ELISA; Fig. 9A through D). There was no difference between the rIBV-vaccinated groups pre-challenge, with all groups higher than the mock-vaccinated groups demonstrating that vaccination had induced an IBV-specific antibody response (Fig. 9B). Post-challenge IBV-specific antibody levels were significantly higher in the M41K-N42A-vaccinated group at 4 dpc (Fig. 9C); however, by 14 dpc, all rIBV-vaccinated groups were comparable (Fig. 9D). The presence and levels of neutralizing antibody were assessed by determining the plaque reduction neutralization titer in serum harvested from each bird (PRNT_50_). No difference between the rIBV-vaccinated groups was observed (Fig. 9E and F).

## DISCUSSION

The coronavirus Mac1 domain has been extensively studied in the context of specific coronaviruses including HCoV 229E, MHV, SARS-CoV, and SARS-CoV-2 all of which infect mammalian host species (48, 54, 55, 58, 59, 61–63, 96, 97). This catalog of research has highlighted a role for the Mac1 domain in the modulation of the mammalian host response to viral infection, specifically the innate immune response and consequently pathogenicity. Our study investigates the Mac1 domain of the *Gammacoronavirus* IBV which infects the avian host species, *Gallus gallus*, commonly referred to as chicken. Sequence alignments of the Mac1 domain from a variety of IBV strains representing a diverse number of genotypes demonstrated a high degree of conservation between IBV strains and the related *Gammacoronavirus* Turkey Coronavirus (Fig. 1). This high level of conservation in a viral pathogen of economic importance and associated food security highlights the Mac1 domain as an attractive target for vaccine development. Our study specifically aimed to identify whether mutations to key residues in the Mac1 domain could be used as a method for rational attenuation of IBV for subsequent vaccine development.

We generated two recombinant IBVs based on the pathogenic M41 strain of IBV, M41K-N42A, and M41K-G49S, with the first containing a Asn→Ala mutation at a highly conserved residue not only within IBV but also the wider coronavirus family, and the second containing a Gly→Ser mutation at the central position within a triple glycine motif that is also highly conserved, particularly within IBV strains and the avian deltacoronaviruses (Fig. 1A and C). The Asn→Ala was chosen due to previous research on SARS-CoV in which it has been demonstrated to reduce the catalytic activity of the Mac1 domain and is associated with attenuation not only for SARS-CoV but also SARS-CoV-2 and MHV (55, 58, 60, 64) (57, 65). The triple Glycine motif that lines the binding cleft has not been previously targeted in pathogenicity research for other coronaviruses; however, we thought it was important to investigate due to the difference in the central amino acid between the attenuated IBV strain Beaudette and the pathogenic M41 strain; a difference that affects structure of the binding pocket (62, 63). The ability to successfully rescue both rIBV M41K-N42A and M41K-G49S alongside the maintenance of the mutations after 10 serial passages suggests neither of these specific mutations to the Mac1 domain was detrimental to IBV replication. This was further supported as neither the Asn→Ala nor Gly→Ser mutation significantly impacted viral replication *in vitro* (Fig. 2). Reduced plaque size was however observed during M41K-N41A infection (Fig. 2C). It must be noted that NGS identified that M41K-G49S contained additional mutations in S and E. To our knowledge, at the time of writing, no interaction has been identified between the Mac1 domain and the structural proteins, S and E. It cannot be ruled out however, that these mutations may in some way be compensatory for the hypothesized impaired function of the Mac1 domain as a result of the G49S mutation. Investigation of the Beaudette sequence (accession number AJ311317), a virus that naturally contains the G49S mutation however does not identify either the S and E mutations present in M41K-G49S suggesting that the acquired mutations may be spontaneous and not related to the G49S mutation in the Mac1 domain.

Previous research has identified no deficiencies in viral replication in cell culture of the equivalent Asn→Ala mutants in HCoV 229E, SARS-CoV, and MHV (58, 60, 61, 64). A recombinant MHV containing the Asn→Ala mutation however did exhibit reduced replication in primary bone-marrow-derived macrophages; this was attributed to sensitivity to innate immune responses (92). Interestingly, reduced plaque size has been associated with sensitivity to IFN responses for other viral pathogens including influenza virus (98) as well as with impacts on viral dissemination, fitness, and virulence for SARS-CoV-2 (99), respiratory syncytial virus (100), and dengue virus (101). As well as reduced plaque size the M41K-N42A exhibited reduced replication in TOCs (Fig. 2F and G) which is hypothesized to be associated with decreased virulence of IBV. Neither *in vitro* nor *ex vivo* did either M41K-N42A or M41K-G49S result in a significant change in the IFN response to viral infection (Fig. 5).

*In vivo* M41K-N42A exhibited reduced pathogenicity both in terms of clinical disease and tracheal ciliary activity (Fig. 3). M41K-G49S also exhibited reduced pathogenicity in terms of clinical disease however tracheal ciliary activity was reduced at 6 dpi, with one bird exhibiting complete ciliostasis, comparable to the M41-K pathogenic control group. Consequently, only can M41K-N42A be considered fully attenuated suggesting that, in line with other coronaviruses, the catalytic activity of the Mac1 domain is a factor in virulence (58, 60, 61, 64). The reduced pathogenic phenotype observed does not appear to be the consequence of reduced viral replication, which is comparable at 4 dpi in tracheal tissue, the primary site of IBV replication (Fig. 3; Table 1). This is in contrast to MHV, SARS-CoV, and SARS-CoV-2 Asn→Ala Mac1 mutants in which reduced pathogenicity was associated with reduced viral load (58–60, 64, 97). Also in contrast, a clear effect on the IFN response, either upregulation or downregulation, was not observed, with changes in IFN expression differing between time point and tissue and not relatable to changes in viral load (Fig. 3 and 4). It is worth noting that all previous research into the coronavirus Mac1 domain and its role in IFN modulation has occurred in mammalian hosts (58, 60, 64); there are differences in IFNs and their receptors and signaling cascades in avian species (102). Research into the IFN response to IBV *in vivo* and *in vitro* has suggested that the responses vary by both strain and time (39, 103–106). There are also other factors encoded in the genome that counteract the host IFN response, such as the accessory proteins 3a and 5b (39, 105, 106). It is therefore possible that mutations to the Mac1 domain have impacted viral sensitivity to the IFN response to infection; however due to the redundancy of this function within the M41 genome, a noticeable effect is not easily observed.

The mechanism by which the Gly→Ser and Asn→Ala mutations to the Mac1 domain are affecting IBV replication and pathogenicity is not entirely clear from our study and requires future research. Our study does highlight differences in the phenotype, *ex vivo, in ovo,* and *in vivo,* of M41K-N42A and M41K-G49S (Fig. 2 and 3). It has been hypothesized that the Gly→Ser mutation in the Mac1 domain may be a factor in the attenuated phenotype exhibited by Beaudette (45, 66). The partially reduced pathogenicity exhibited by M41K-G49S may support an involvement of the Mac1 in the attenuated phenotype of Beaudette. As we believe this is the first account of the triple glycine motif being targeted for mutagenesis, it remains to be seen if this motif affects the pathogenicity of other coronaviruses. Previous research has identified phenotypic differences between recombinant coronaviruses containing different mutations to the Mac1 domain (58, 96, 97).

The primary aim of our study was not to elucidate function but to identify genome targets for rational attenuation for vaccine development with an additional aim of assessing the effects on vaccine efficacy of combining multiple attenuating mutations into one vaccine virus. As defined by tracheal ciliary activity M41K-N42A exhibited an attenuated phenotype on both 4 and 6 dpi, we therefore incorporated the Asn→Ala mutation into the rIBV M41-R generating M41R-N42A (Fig. 6). M41-R was generated during the development of a reverse genetics system based on the virulent M41 strain of IBV and was found to be attenuated (41). The attenuated phenotype was linked to four amino acid changes Pro85Leu in Nsp10, Val393Leu in Nsp14, Leu183Ile in Nsp15, and Val209Ile in Nsp16, of which a determining role was highlighted for Pro85Leu in Nsp10 and Val393Leu in Nsp14 (41). Our previous research demonstrated that not only did the two changes in Nsp10 and 14 impart an attenuated phenotype, but also a *ts* replication phenotype, and additionally vaccination with M41-R can protect against homologous challenge (41, 42). While the changes in Nsp15 and 16 are not thought to play a role in the *ts* replication phenotype nor pathogenicity, they may contribute to the stability of M41-R as no changes were observed upon serial passage (41). Replication of M41R-N42A was largely comparable to the parental M41-R (Fig. 6). Unlike M41K-N42A, there was no reduction of M41R-N42A replication *in ovo* with titers exceeding 10^6^ PFU/mL comparable to both M41-K and M41-R (Fig. 2H and 6G). This may suggest a synergic effect of the mutations and interestingly *ts* mutants of MHV with mutations in the Mac1 domain have indicated a possible interaction with Nsp10 (107). It must be noted NGS sequencing for M41R-N42A highlighted the presence of two nonsynonymous mutations in S and a two-nucleotide deletion in the putative accessory gene 4b (7). It remains to be determined while these mutations have not impacted vaccine efficacy (discussed more below) whether they have impacted replication *in ovo* or whether the observed effect is in fact the result of the combination of the mutations in Nsp10, 14 15, 16 with the N42A mutation in the Mac1 domain. Regardless of the mechanism the *in ovo* phenotype observed is beneficial in terms of vaccine development as current vaccine manufacturing practices rely on virus propagation in embryonated hens’ eggs.

Both M41K-N42A and M41R-N42A were able to protect against homologous challenge as defined by the absence of clinical disease post-challenge, retention of tracheal ciliary activity, and the absence of challenge virus (Fig. 7; Table 2). Both M41K-N42A and M41R-N42A alongside the previously characterized M41-R (42) meet the standards set by the European Pharmacopoeia (108). In addition, the lack of infectious challenge viral progeny and in some birds the absence of challenge viral RNA indicates that vaccination with rIBV M41-R, M41R-N42A, or M41K-N42A is sufficient to elicit an immune response that can prevent productive challenge virus replication. There was no difference between the protection offered suggesting that the combination of attenuating mutations in Nsp3 (N42A), Nsp10 (Pro85Leu), and Nsp14 (Val393Leu) does not negatively impact vaccine efficacy. This is a promising step for vaccine development as distally located mutations spread along the genome may reduce the chance of recombination events leading to a virulent outcome. In addition, it also reduces the risk of reversion to virulence; a risk that is already minimized due to the clonal origin of the rIBVs. The Asn42 residue in Nsp3 alongside Pro85 in Nsp10 and the Val393 in Nsp14 are conserved not only among IBV strains but also in the wider coronavirus family and therefore may offer an avenue for the development of a broad range of coronavirus vaccines.

## Data Availability

All experimental data required for the evaluation of the conclusions of this study are presented within this paper. All genome sequences used for the evaluation of the conservation of the Mac1 domain are publicly available with accession numbers provided in the relevant sections.
